# Laser Doppler Gas Flowmeter with Synchronous Three-Point Measurement

**DOI:** 10.3390/s26185765

**Published:** 2026-09-10

**Authors:** Jian Zhou, Bolin Li, Shuang Zhang, Xiaoming Nie

**Affiliations:** 1 College of Advanced Interdisciplinary Studies, National University of Defense Technology, Changsha 410073, China; 2 Nanhu Laser Laboratory, National University of Defense Technology, Changsha 410073, China

**Keywords:** laser Doppler velocimetry, gas flow rate measurement in pipelines, three-point synchronous measurement, velocity profile fitting

## Abstract

To address the challenges of susceptibility to interference and limited accuracy inherent in conventional gas flow rate measurement methods, this paper proposes and investigates a laser Doppler gas flow rate measurement method based on synchronous three-point velocity measurement. This method simultaneously measures the flow velocities at three characteristic points within the pipeline cross-section, subsequently fits and reconstructs the velocity distribution across the entire profile, and ultimately achieves high-precision flow measurement. The feasibility of selecting the center point, the quarter-width point, and the near-wall point as the three characteristic measurement positions is analyzed through computational fluid dynamics simulations, and the full-profile velocity distribution is fitted accordingly. A three-point synchronous velocity-flow rate measurement system is designed and constructed, employing a transmitting optical path based on the “three beam splitters and two mirrors” scheme and a receiving optical path based on the “multi-lens independent reception” scheme, and experimental validation is conducted. Experimental results demonstrate that the system operates stably with good repeatability. In contrast to the flow calculation method using a single-point Pitot tube combined with an empirical formula, the proposed system, which directly fits the velocity profile and integrates it for flow calculation, effectively avoids the significant model errors caused by using fixed empirical coefficients in non-circular pipe flows, and is inherently more universally applicable in principle. Through comparison with the TSI reference standard (3D LDV), the measurement results of the proposed system are in close agreement with the reference values, with relative errors all below −0.8%. This paper provides an effective solution for high-precision gas flow measurement in square pipelines.

## 1. Introduction

Pipeline gas flow rate measurement holds irreplaceable strategic value across multiple critical sectors, serving as a fundamental foundation for ensuring efficient resource utilization and sustainable development. In recent years, gas flow rate measurement technologies have undergone significant advancements, giving rise to various instruments and devices based on different principles, such as the Pitot tube based on differential pressure, electromagnetic flowmeters based on electromagnetic principles, vortex flowmeters based on the Kármán vortex street principle, turbine flowmeters based on volumetric principles, and ultrasonic flowmeters based on the acoustic Doppler principle.

However, each device possesses inherent limitations (Table 1). For instance, the Pitot tube requires a long straight pipe section to ensure measurement accuracy and the small differential pressure at low flow rates leads to poor accuracy [1,2]; electromagnetic flowmeter can only measure conductive liquids, and is unable to measure oil, pure water or gases [3,4]; vortex flowmeter has its lower measurement limits constrained by factors such as Reynolds number, vortex energy, and temperature [5,6]; turbine flowmeter has mechanical moving parts and is prone to interference during actual measurements, resulting in suboptimal accuracy [7,8]; and ultrasonic flowmeter imposes relatively high demands on medium purity. Moreover, its measurement accuracy can be compromised by the pipe wall material and scaling, and the device is generally unsuitable for non-metallic pipes [9,10,11].

Given the inherent shortcomings of conventional flow rate measurement methods, the application of laser Doppler measurement techniques for fluid flow measurement presents a comparatively ideal alternative. Relevant research has been conducted by institutions such as Tsinghua University [12], Northwestern Polytechnical University [13], and the National Petroleum and Natural Gas Large Flow Measurement Station [14]. Nevertheless, existing technical approaches still face two major challenges [15,16,17]. First, single-point velocity measurements within a cross-section cannot accurately reflect the overall velocity distribution across the entire profile, leading to significant errors when inferring flow rate from a single point. Second, while one-dimensional scanning of a single point can yield axial velocity profiles, or two-dimensional scanning can map the cross-sectional velocity field--thereby improving measurement accuracy--such approaches are excessively time-consuming and suffer from asynchrony in velocity measurements at different spatial positions. To address these issues, this paper proposes a low-complexity, high-precision, and fast flow reconstruction method based on synchronous multipoint measurement with Laser Doppler velocimetry technology.

## 2. Selection and Analysis of Measurement Points

For square pipeline, Lu Zhanmin pointed out in his experimental study that for flows with precise geometric dimensions, uniform inlet conditions, and fully developed characteristics, the flow field is generally symmetric about two orthogonal planes that pass through the center of the cross-section and are parallel to the side walls. He also found that the velocity distribution in a square duct is more “flat” and tends strongly toward greater uniformity with increasing Reynolds number [18]. For the fully developed turbulent flow in a square pipeline, this paper establishes a numerical simulation model using the Computational Fluid Dynamics (CFD) method. A square pipeline was selected for this study for two primary reasons. One is that the turbulent velocity distribution and flow structures in square ducts are more complex and distinctive than those in circular pipes, offering greater research value [19]. The other is that, in terms of experimental implementation, LDV requires optical access through transparent windows, which are easier to fabricate and mount on planar surfaces in square ducts, simplifying the optical setup and velocity measurements.

**Figure 1:**
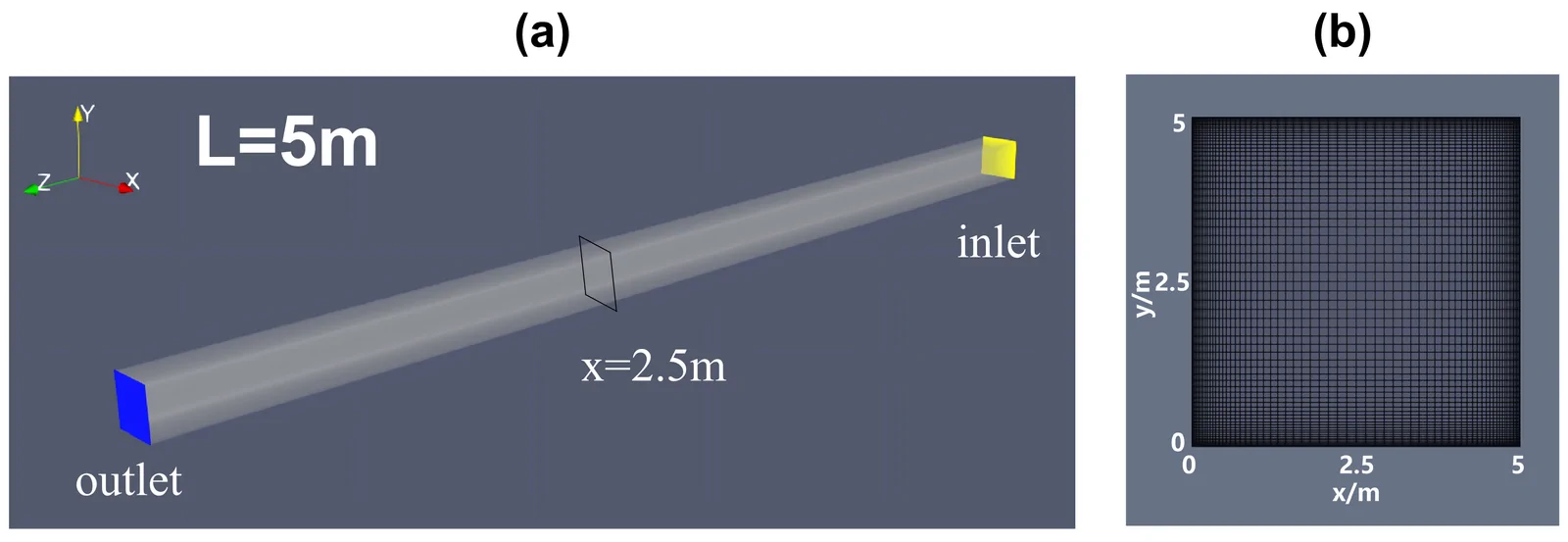
Simulation settings (**a**) simulation flow field calculation domain; (**b**) schematic diagram of simulation grid division.

In accordance with practical flow measurement applications, a computational domain was constructed as illustrated in Figure 1a. The pipeline has a square cross-section with a side length of 0.2 m; the yellow and blue planes represent the inlet and outlet, respectively. The total axial length of the pipeline was set to 5 m (a typical length in real applications) to ensure that the flow is fully developed at the measurement plane and to minimize the influence of the inlet and outlet recirculation zones. The measurement plane was chosen at the mid-length of the pipeline (x = 2.5 m), sufficiently downstream of the inlet to guarantee a fully developed turbulent state. A near-wall mesh refinement strategy was adopted to resolve the velocity boundary layer with sufficient accuracy (Figure 1b). The grid density in the near-wall region was set to six times that in the core flow region. The total number of grids is 5184. This approach ensures that the steep velocity gradients adjacent to the wall are properly captured, thereby avoiding numerical errors that may result from an overly coarse mesh in this critical zone.

Standard air (the density $\rho =1.225\;{\mathrm{kg}/m}^{3}$, the dynamic viscosity $\eta =1.7894\times 10^{-5}\;\mathrm{Pa}\cdot s$), modeled as an incompressible viscous fluid, is used as the working medium in the computational domain. The flow field is governed by the Reynolds-averaged Navier–Stokes (RANS) equations:

(1) $$\frac{\partial u_{i}}{\partial x_{i}}=0$$

(2) $$\rho \frac{\partial {\bar{u}}_{i}}{\partial t}+\rho {\bar{u}}_{j}\frac{\partial {\bar{u}}_{i}}{\partial x_{j}}=\rho {\bar{F}}_{i}-\frac{\partial \bar{p}}{\partial x_{i}}+\frac{\partial }{\partial x_{j}}\left(\mu \frac{\partial {\bar{u}}_{i}}{\partial x_{j}}-\rho \bar{u_{i}^{\prime }u_{j}^{\prime }}\right)$$

where $u_{i}$ is the velocity component along the $x_{i}$ direction in the Cartesian coordinate system (i,j=1,2,3), ${\bar{u}}_{i}$ is the time-averaged velocity, $\bar{p}$ is the mean fluid pressure, ${\bar{F}}_{i}$ is the mean body force acting on the fluid, ρ is the fluid density, μ is the fluid velocity, $u_{i}^{\prime }$ is the fluctuating velocity, and $-\rho \bar{u_{i}^{\prime }u_{j}^{\prime }}$ is the Reynolds stress, which accounts for the effect of turbulent fluctuations on the mean flow. To close the governing equations, a constitutive relation between the Reynolds stress and the mean velocity gradient must be established.

The modeling of Reynolds stress is central to turbulence simulation. In this paper, the standard k-ω model is adopted [20]. This model offers advantages in simulating wall-bounded flows and handling low-Reynolds-number effects in the near-wall region, thereby meeting the requirement of accurately capturing the velocity gradient in the near-wall region for the present study. The standard k-ω model is a two-equation eddy-viscosity model. It can be integrated directly into the wall without the use of wall functions in the near-wall region, and exhibits good robustness in simulating complex flow phenomena such as adverse pressure gradients. Consequently, it is well-suited for resolving the turbulent boundary layer in a square duct, where significant wall effects and corner flows are present. Its governing equations are as follows:

(3) $$\frac{\partial (\rho k)}{\partial t}+\frac{\partial (\rho {\bar{u}}_{j}k)}{\partial x_{j}}=\frac{\partial }{\partial x_{j}}\left[\left(\mu +\frac{\mu_{t}}{\sigma_{k}}\right)\frac{\partial k}{\partial x_{j}}\right]+P_{k}-\rho \beta^{*}k\omega$$

(4) $$\frac{\partial (\rho \omega )}{\partial t}+\frac{\partial (\rho {\bar{u}}_{j}\omega )}{\partial x_{j}}=\frac{\partial }{\partial x_{j}}\left[\left(\mu +\frac{\mu_{t}}{\sigma_{\omega }}\right)\frac{\partial \omega }{\partial x_{j}}\right]+\alpha \frac{\omega }{k}P_{k}-\rho \beta \omega^{2}$$

(5) $$\mu_{t}=\rho \frac{k}{\omega }$$

where $\mu_{t}$ is coefficient of eddy viscosity; $P_{k}$ is turbulent kinetic energy generation term; the dissipation term coefficient of the k equation $\beta^{*}=0.09$; the generated term coefficient of the ω equation α=0.52; the dissipation term coefficient of the ω equation β=0.072; the diffusion term coefficient of the k equation $\sigma_{k}=2.0$; the diffusion term coefficient of the ω equation $\sigma_{\omega }=2.0$.

The inlet boundary was set as a velocity inlet, with four velocities (1 m/s, 3 m/s, 5 m/s, and 8 m/s) selected within the typical measurement range of 1–10 m/s, corresponding to different steady flow conditions. The outlet boundary was specified as outflow to represent unconstrained flow conditions, and all wall boundaries were assigned no-slip conditions. The convergence criterion was defined as all residuals falling below 10^−5^ in magnitude while the monitored velocity at the probe point remained stable. Based on the converged steady-state flow field, the full-field velocity distribution at the cross-section x = 2.5 m was extracted to serve as the data foundation for flow field characterization and measurement method validation.

**Figure 2:**
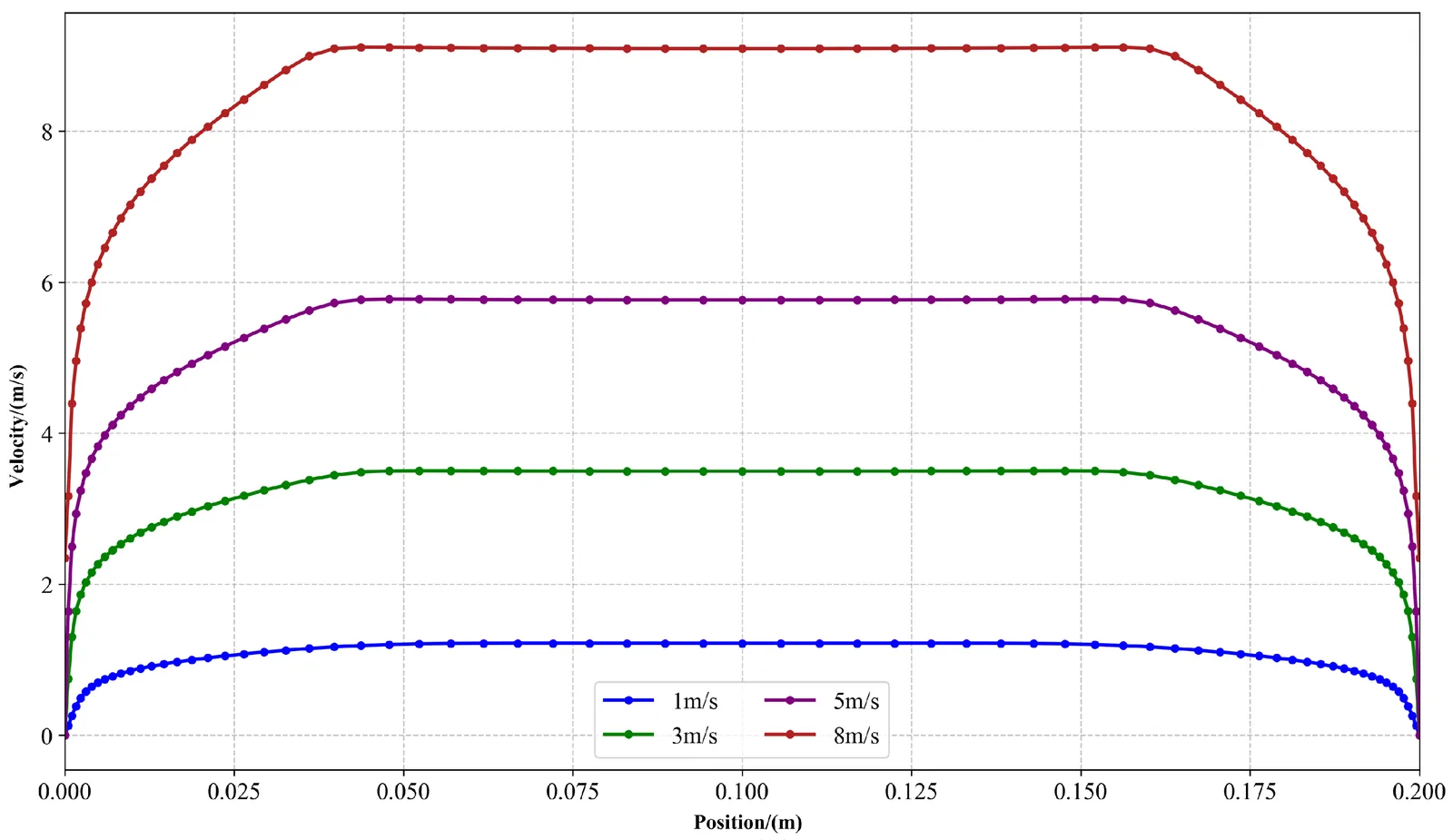
The velocity distribution curves along the horizontal straight line at the center of the pipe under inlet speeds of 1 m/s, 3 m/s, 5 m/s and 8 m/s, respectively.

Simulation analyses of the velocity distribution at the middle cross-section of the pipeline were conducted under inlet flow velocities of 1 m/s, 3 m/s, 5 m/s, and 8 m/s, with the results presented in Figure 2. The results indicate that under different flow velocities, the centerline velocity profiles all display a distinct three-zone structure comprising the “core region, transition region, and near-wall region.”

**Figure 3:**
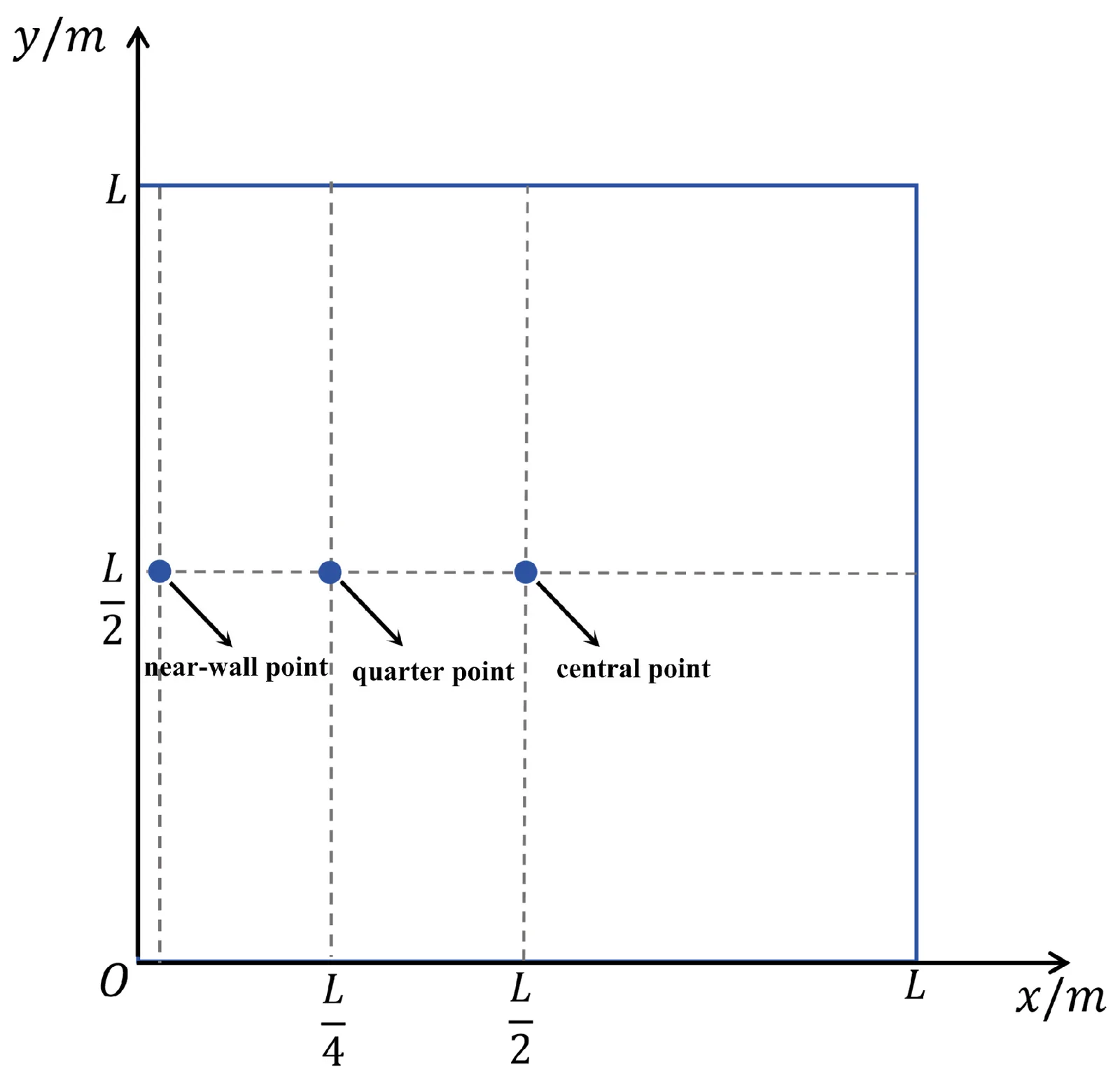
Three-point scheme diagram.

To further investigate the influence of the number of measurement points on flow measurement accuracy, a simulation analysis was conducted under the condition of 1 m/s. Based on the symmetry of the velocity distribution, virtual measurement point groups ranging from 2 to 7 points were arranged within the half-width region of the pipeline (0 ≤ x ≤ 0.1 m). All measurement point groups explicitly include both the center point and the near-wall point, with the remaining points uniformly distributed within this region, thereby ensuring comprehensive coverage and uniform characterization of the velocity variation trend across the entire half-width range. Among them, the three-point scheme selects one point from each of the core region, transition region, and near-wall region, namely the center point, the quarter-width point, and the near-wall point, as shown in Figure 3.

**Figure 4:**
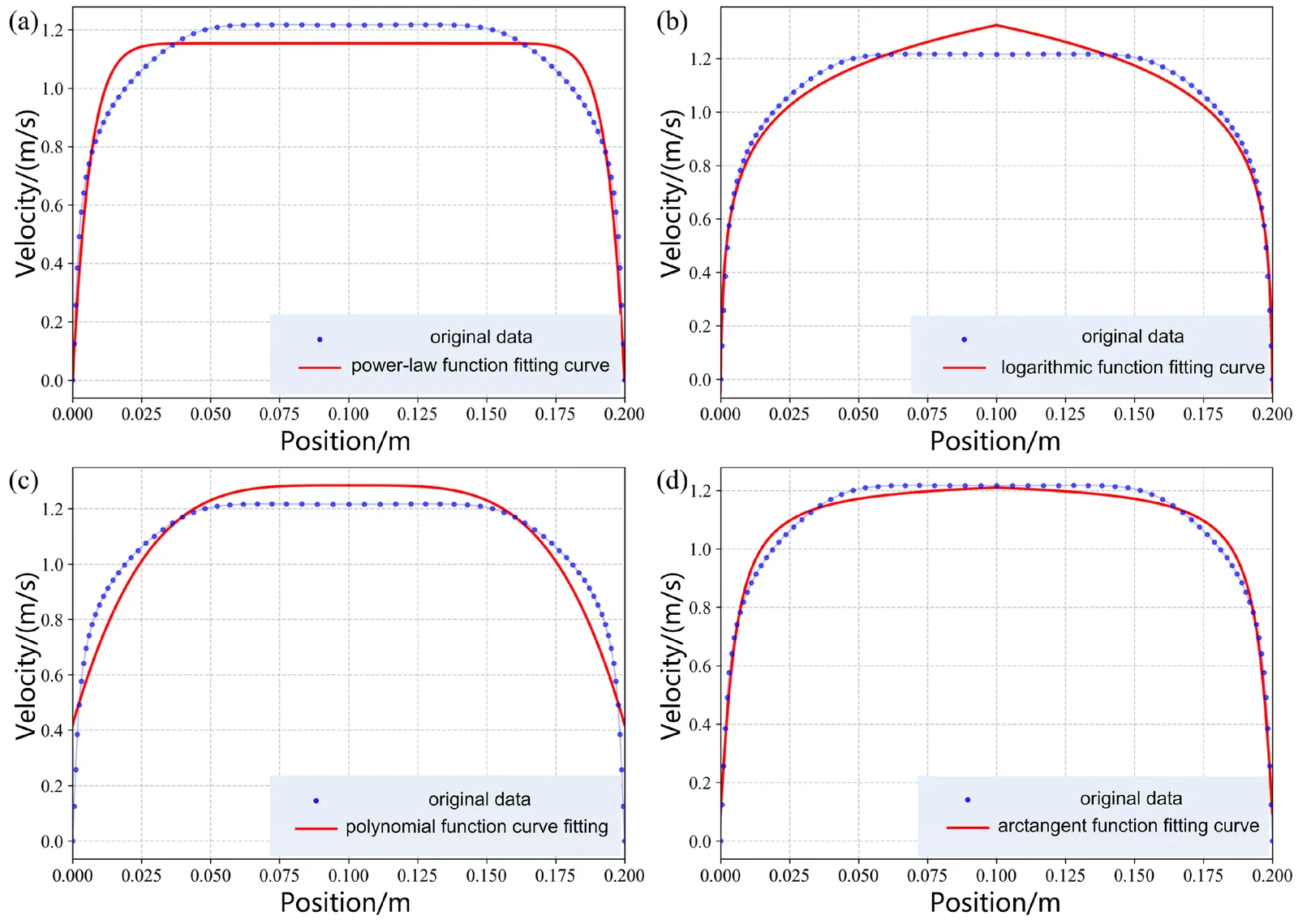
Comparison chart of different fitting functions and CFD simulation velocity distribution (**a**) power-law function; (**b**) logarithmic function; (**c**) polynomial function; (**d**) arctangent function.

To mathematically determine the optimal functional form for fitting the velocity profile, a quantitative comparison among different candidate functions was conducted. As established in the preceding analysis, the velocity distribution of fully developed turbulent flow in a square duct exhibits a similar three-layer structure—consisting of a core region, a transition region, and a near-wall region—across different flow velocities, indicating a self-similar nature of the profile shape. Therefore, the case with an inlet velocity of 1 m/s was selected as a representative condition for in-depth analysis. Figure 4 compares the CFD-predicted velocity distribution with the fitting curves from four candidate functions under a representative condition. The fitting performance of the candidate functions is quantitatively compared in Table 2.

From the results in Figure 4 and Table 2, several observations can be made. The power-law function gives an R^2^ of 0.949 but a relatively large MRE of 9.39%, indicating that while it captures the general trend, it deviates considerably in local regions—especially in the transition zone. The logarithmic-law function performs better overall (R^2^ = 0.976, MRE = 7.74%), yet still shows noticeable deviations in the core region. The polynomial function, with an MRE as high as 17.10%, lacks the flexibility required to describe the velocity profile over the entire range. By contrast, the arctangent function achieves the highest R^2^ (0.986) and the lowest MRE (4.69%), demonstrating both the best agreement with the true profile and the highest overall accuracy. The fitted curves are in good agreement with the simulated data, confirming the superior performance of this function. Based on these quantitative comparisons, the arctangent function clearly outperforms all other candidates. It is therefore selected as the mathematical model for velocity profile fitting and subsequent flow rate determination.

**Figure 5:**
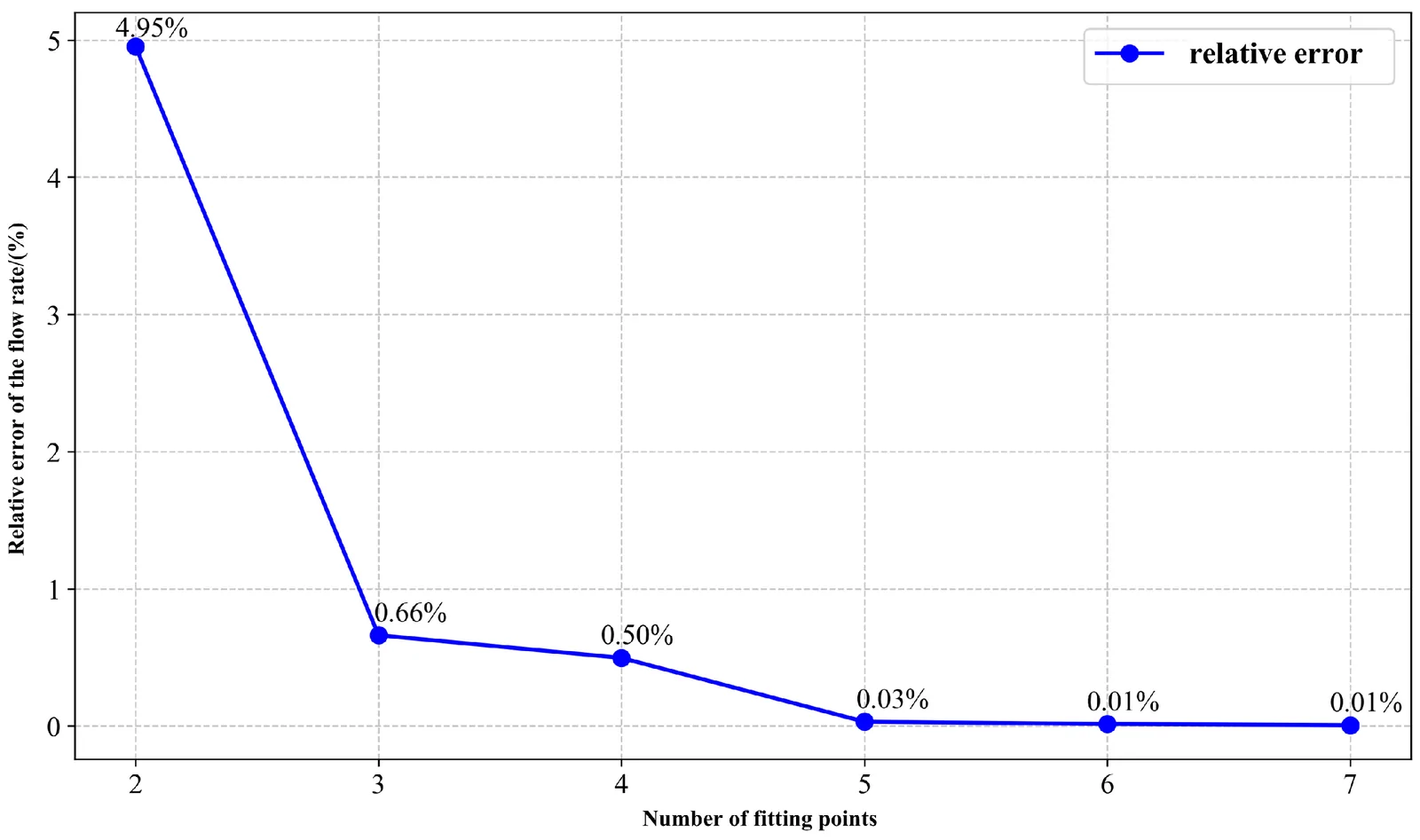
Relative error for different numbers of fitting points.

For all measurement point schemes, the arctangent function was employed for fitting and flow integration, and the relative error with respect to the CFD standard flow rate was calculated. The relationship between the number of fitting points and the relative error of flow rate calculation is shown in Figure 5.

As shown in Figure 5, the flow calculation error decreases monotonically with an increasing number of measurement points, which is consistent with theoretical expectations. However, the marginal gain of error reduction diminishes sharply. Specifically, when the number of measurement points increases from 2 to 5, the absolute error decreases from 4.95% to 0.03%, representing a significant improvement in accuracy; whereas when the number increases from 5 to 7, the error only drops from 0.03% to 0.01%, yielding negligible further improvement. This clearly indicates that with only three strategically positioned measurement points, the majority of the accuracy benefits can already be achieved. Considering that in engineering practice, each additional measurement point entails increased optical complexity, hardware cost, commissioning difficulty, and system instability, the three-point measurement scheme not only meets the engineering requirements for flow measurement accuracy but also offers relatively lower optical implementation complexity and system complexity. It thus represents an appropriate choice that balances measurement accuracy and engineering practicality.

## 3. Principle of Laser Doppler Gas Flow Rate Measurement

The optical configuration of laser Doppler velocimetry systems generally falls into three primary categories: the reference-beam mode, the self-mixing mode, and the dual-beam differential mode, each possessing distinct characteristics [21]. Given that the present system is intended for gas flow rate measurement within pipelines, where the transverse velocity component is the primary target, and considering the high precision required for accurate flow rate determination, the selection of an appropriate optical arrangement is critical. The reference-beam mode is better suited for longitudinal velocity measurements, whereas the self-mixing mode suffers from insufficient accuracy due to its significant sensitivity to Doppler frequency measurement errors induced by fluctuations in operating current and ambient temperature. Consequently, the dual-beam differential mode is adopted as the fundamental optical configuration in this study.

### 3.1. Dual-Beam Differential Laser Doppler Velocimetry Technique

**Figure 6:**
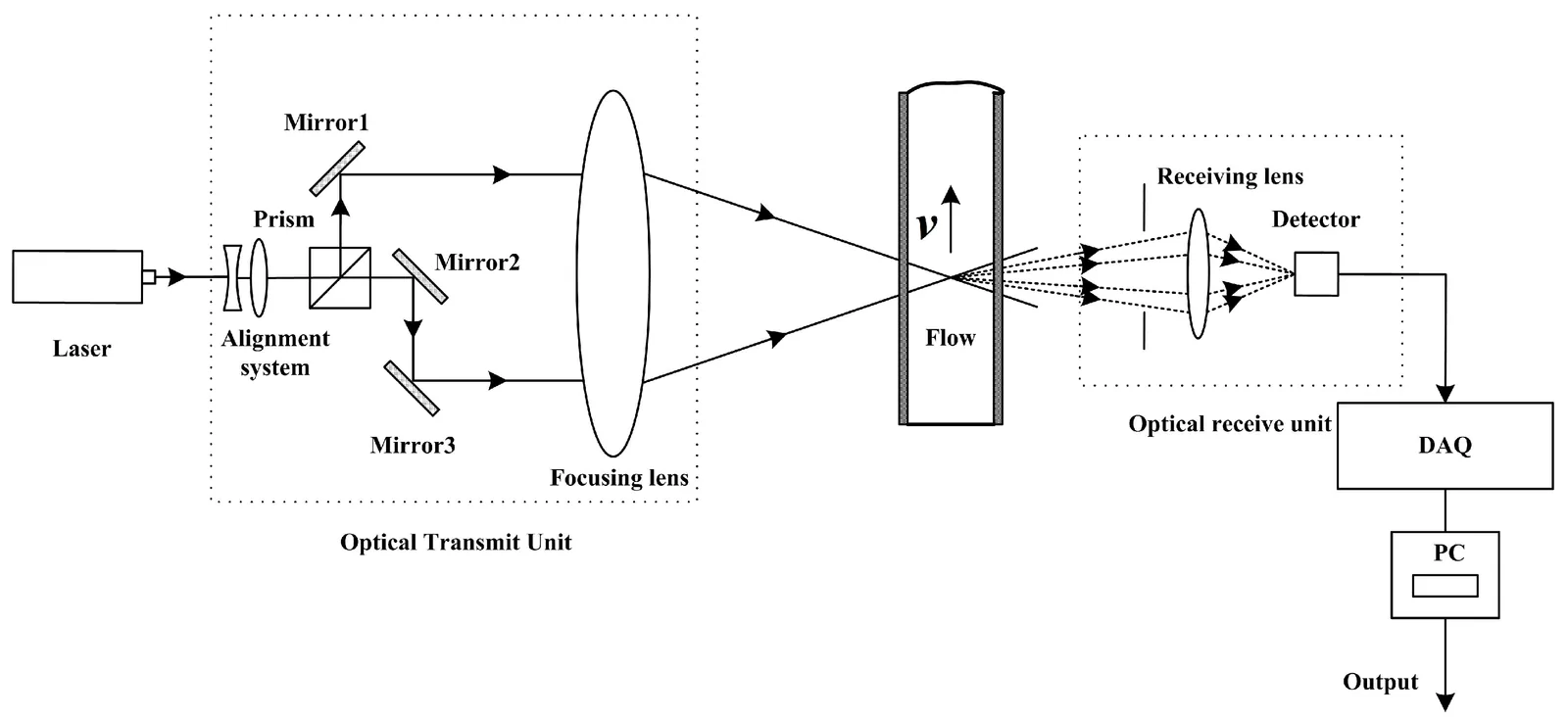
Schematic diagram of the core module of the dual-beam differential Laser Doppler Velocimetry system.

In view of the high transmittance of the fluid, the forward-scattering configuration yields a more intense signal light. Accordingly, the core modules of the system are designed on the basis of the dual-beam differential forward-reception mode, as illustrated in Figure 6.

The core module consists of three parts: the transmitting unit, the receiving unit, and the signal processing unit. The laser beam emitted from the laser source passes through a collimation system and is then split into two beams by a beam splitter. Beam 1 is reflected by mirror 1, while beam 2 is reflected by mirrors 2 and 3, resulting in two parallel beams. These two beams are then focused by a focusing lens to intersect at a measurement volume within the pipeline, where the tracer particles in the gas flow pass through the focal point of the lens. Since the gas flow has a transverse velocity component and the gas is optically transparent, most of the scattered light transmits through the flow in the forward direction, passes through an aperture, and is collected by the receiving lens onto a photodetector. The photodetector converts the received optical signal into an electrical signal, which is then acquired by a data acquisition card and transmitted to a PC. An upper-computer software program extracts the Doppler frequency from the acquired signal, and the flow velocity is ultimately calculated based on the extracted Doppler frequency. The relationship between the measured velocity and the Doppler frequency is given by [21]:

(6) $$v=\frac{f_{D}\cdot \lambda }{2\sin\frac{\theta }{2}}$$

where $f_{D}$ is the Doppler frequency; λ is the wavelength; θ is the angel of the two converging beams.

### 3.2. Three-Point Profiling Flow Rate Measurement System

Considering that a single-point velocity within the cross-section cannot accurately reflect the overall velocity distribution across the entire profile, and targeting the issue of gas flow rate measurement in square pipelines, this paper proposes an indirect measurement method based on profile reconstruction using limited-point velocity data. Three critical positions along the same straight line within the half-width region of the pipeline cross-section are selected for synchronous velocity measurement, namely the center point, the quarter-width point, and the near-wall point. The velocity distribution is then reconstructed based on these measurements, from which the flow rate is subsequently calculated.

**Figure 7:**
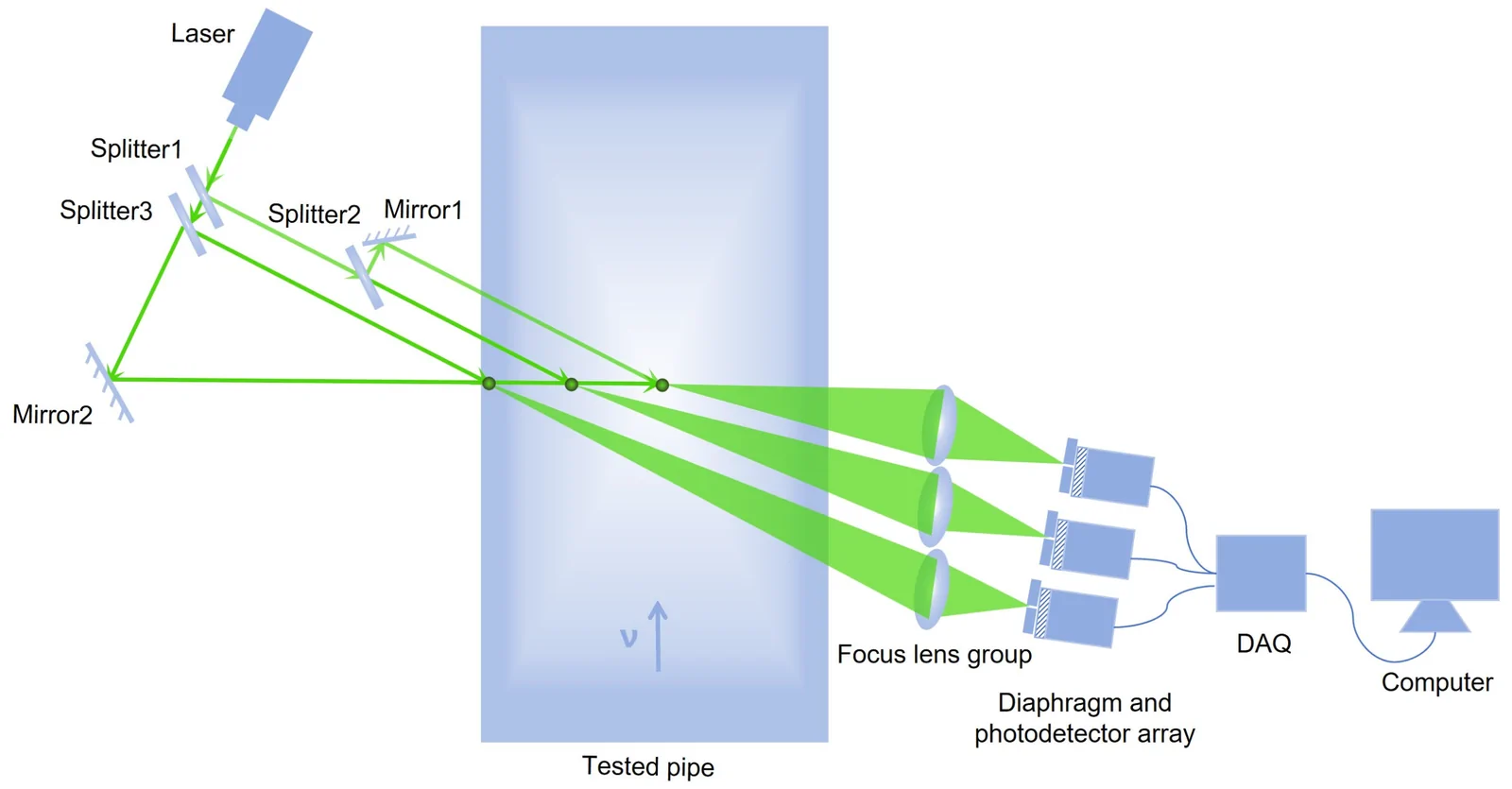
Structural block diagram of the Laser Doppler gas flow rate measurement system.

Considering that the measurement points of the entire system are not a single point but three points, this paper appropriately simplifies the system structure based on the core module of the dual-beam differential laser Doppler velocimetry system. The optimized system block diagram is shown in Figure 7.

The transmitting optical unit is constructed based on a “three beam splitters and two mirrors” scheme. The laser beam emitted from the laser source sequentially passes through three 1:1 beam splitters and two mirrors, ultimately generating three mutually parallel beams and one tilted beam with a specific angle. By finely adjusting the overall orientation of the transmitting unit, the tilted beam and the three parallel beams are made to intersect successively within the same cross-section of the test pipeline, thereby producing three spatially fixed measurement volumes (i.e., the three beam intersection points) within the half-width region of the pipeline.

The receiving optical unit adopts a “multi-lens independent reception” scheme. On the opposite side of the pipeline, three identical focusing lenses are placed in one-to-one correspondence with the three measurement volumes, each collecting the scattered light generated by tracer particles at its respective measurement volume. Each lens focuses the collected scattered light onto the photosensitive surface of an independent avalanche photodiode. The electrical signals output from the three detectors are synchronously acquired by a data acquisition card and transmitted to a computer for subsequent signal processing.

### 3.3. Signal Acquisition and Processing

To extract accurate velocity information from the raw voltage signals and precisely calculate the instantaneous flow rate, this paper designs a complete data processing algorithm. The algorithm converts the voltage signals from the three measurement points into reliable instantaneous flow rate values through spectral analysis, velocity calculation, profile fitting, and integration.

**Figure 8:**
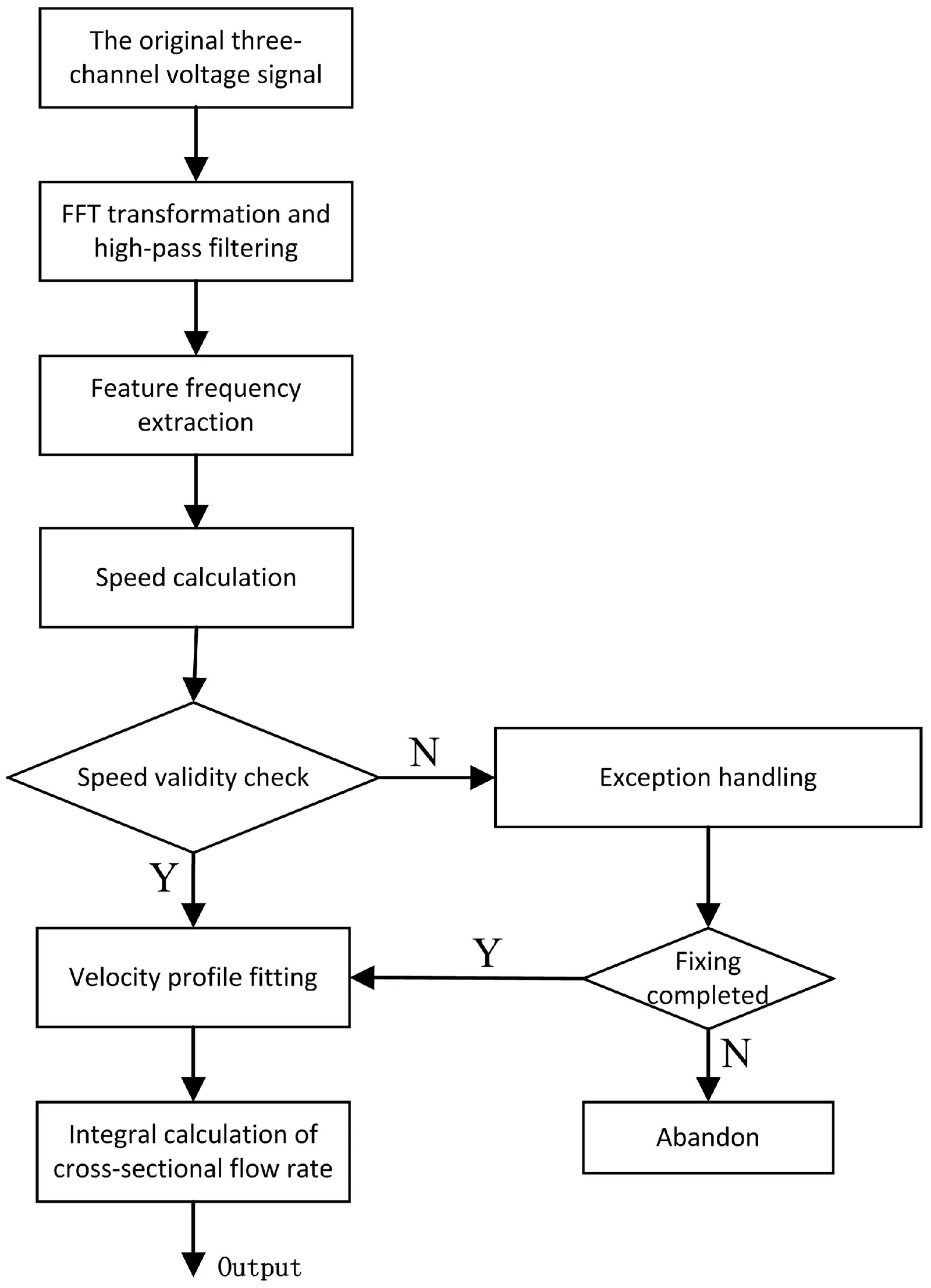
Flowchart of the signal acquisition and processing algorithm.

The overall flow of the algorithm is illustrated in Figure 8. First, fast Fourier transform (FFT) is applied to the three acquired voltage signals to convert them into the frequency domain, followed by high-pass filtering to further eliminate residual low-frequency environmental vibration and noise interference. Subsequently, the characteristic frequencies at the three points are extracted. After obtaining the Doppler frequencies at the three points, the velocities are calculated according to Equation (4), and the reasonableness of the three velocity values is synchronously verified and corrected. For a fully developed turbulent flow, the velocity profile can be well described by an arctangent function model. In this paper, the velocity profile fitting is performed based on the selected arctangent function model, with the model parameters determined by the least-squares method using the measured values at the three characteristic points. Finally, the instantaneous volumetric flow rate at that moment is obtained by integrating the fitted velocity profile over the entire pipeline cross-section.

## 4. Experimental Results and Analysis

To verify the flow rate measurement performance of the proposed synchronous three-point LDV system, tests were conducted in the BLWT-200/200 DC wind tunnel manufactured by Bailin Technology (Suzhou, China). Its main technical parameters are shown in Table 3.

**Figure 9:**
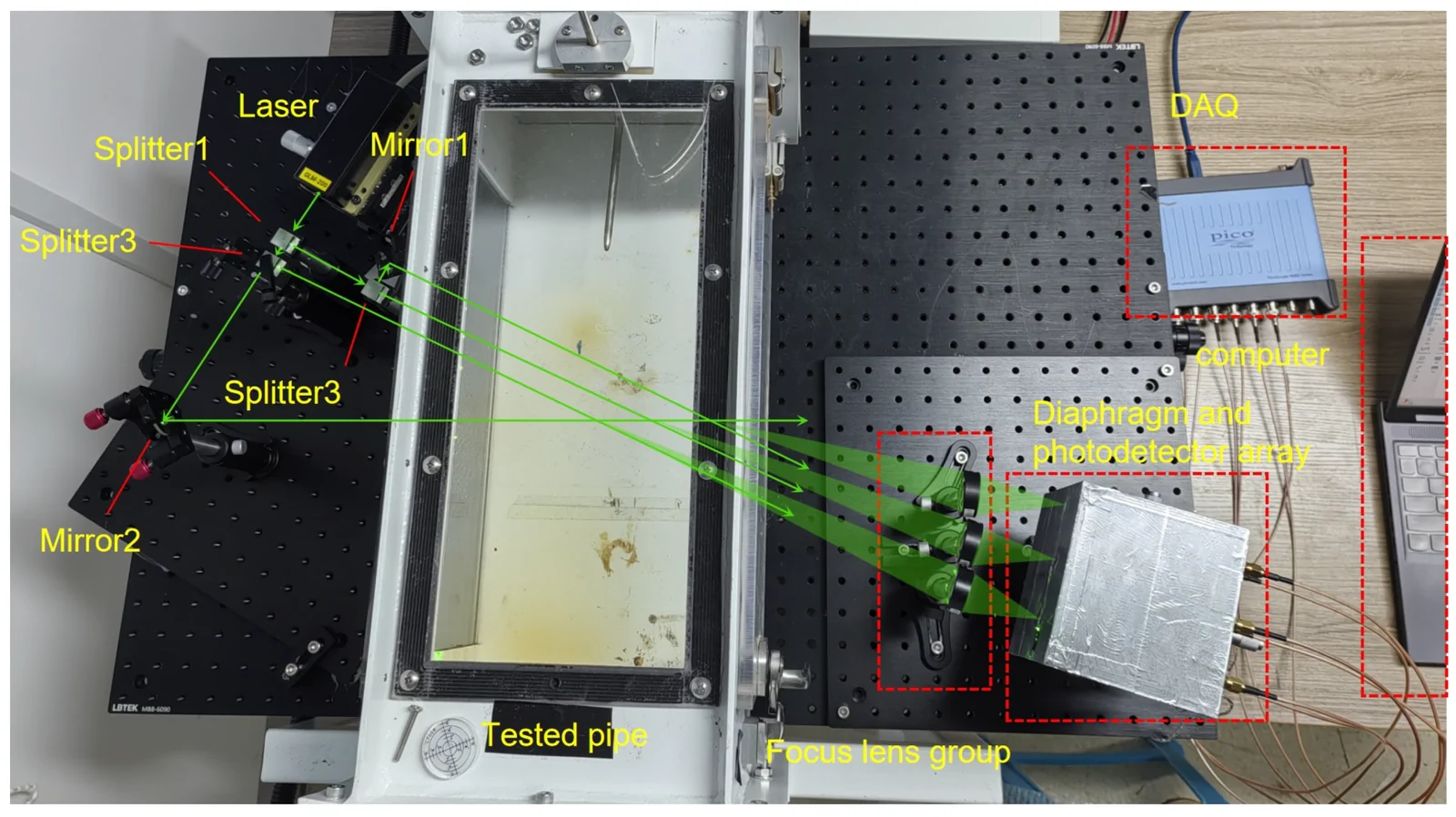
Photograph of the three-point synchronous LDV measurement system.

The overall optical configuration of the system, constructed according to the block diagram shown in Figure 7, is presented in Figure 9. The core of this configuration lies in generating three measurement volumes at distinct spatial positions within the same cross-section of the test pipeline, enabling synchronous measurement at the three characteristic points: the center point, the quarter-width point, and the near-wall point of the pipeline cross-section.

**Figure 10:**
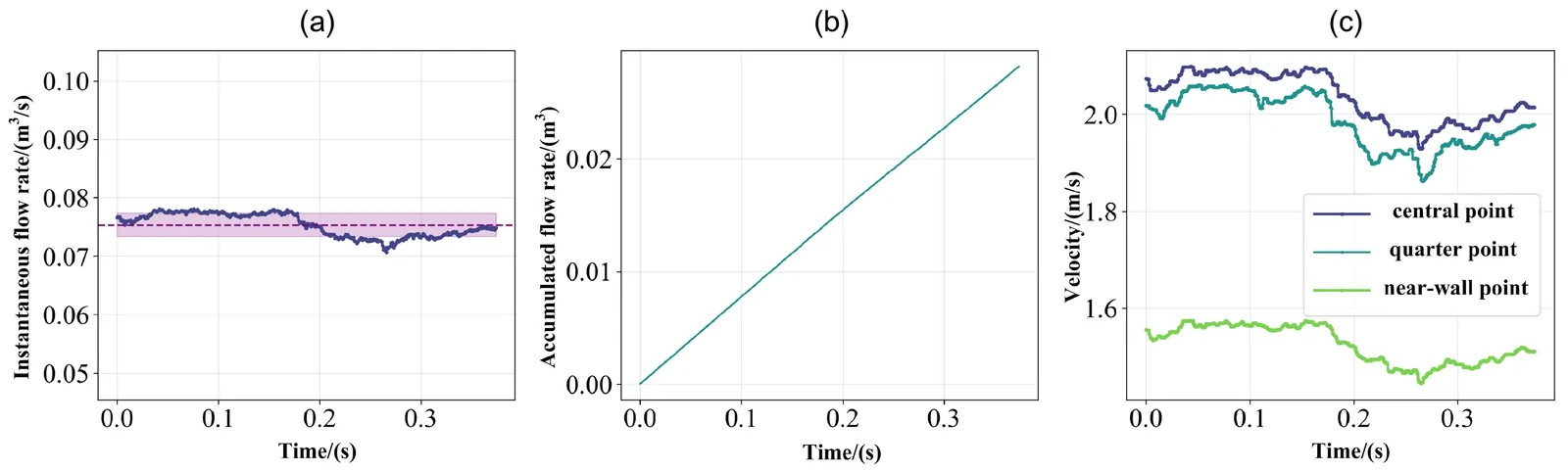
Test results of 1000 consecutive frames at 2 m/s (**a**) the instantaneous flow rate curve; (**b**) the accumulated flow rate curve; (**c**) the velocity variation curves at each measurement point.

**Figure 11:**
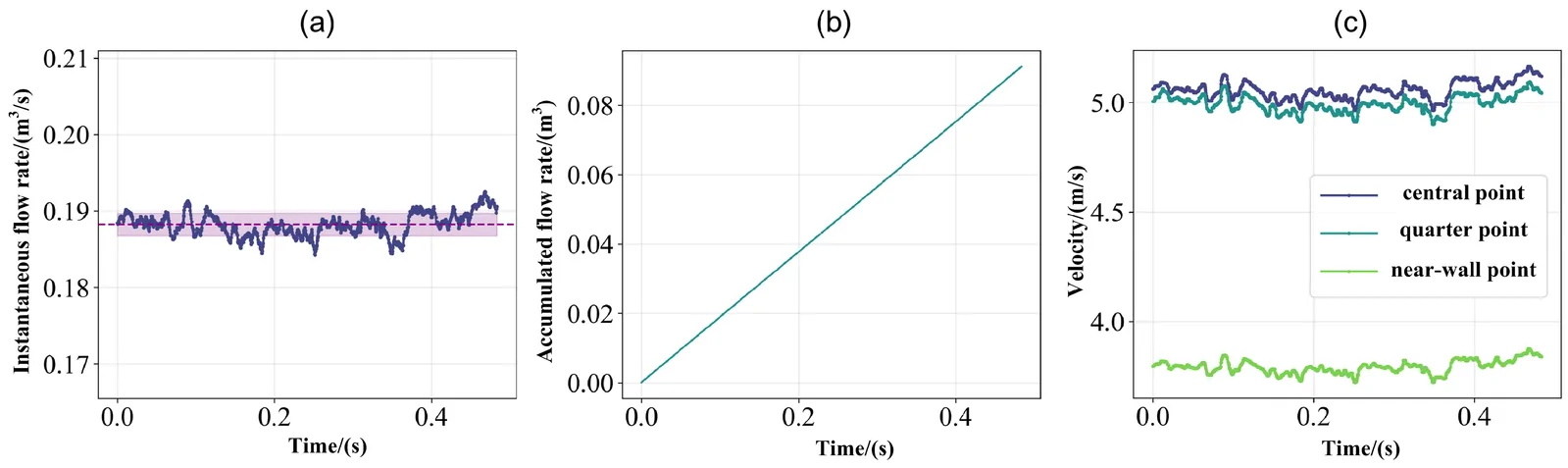
Test results of 1000 consecutive frames at 5 m/s (**a**) the instantaneous flow rate curve; (**b**) the accumulated flow rate curve; (**c**) the velocity variation curves at each measurement point.

**Figure 12:**
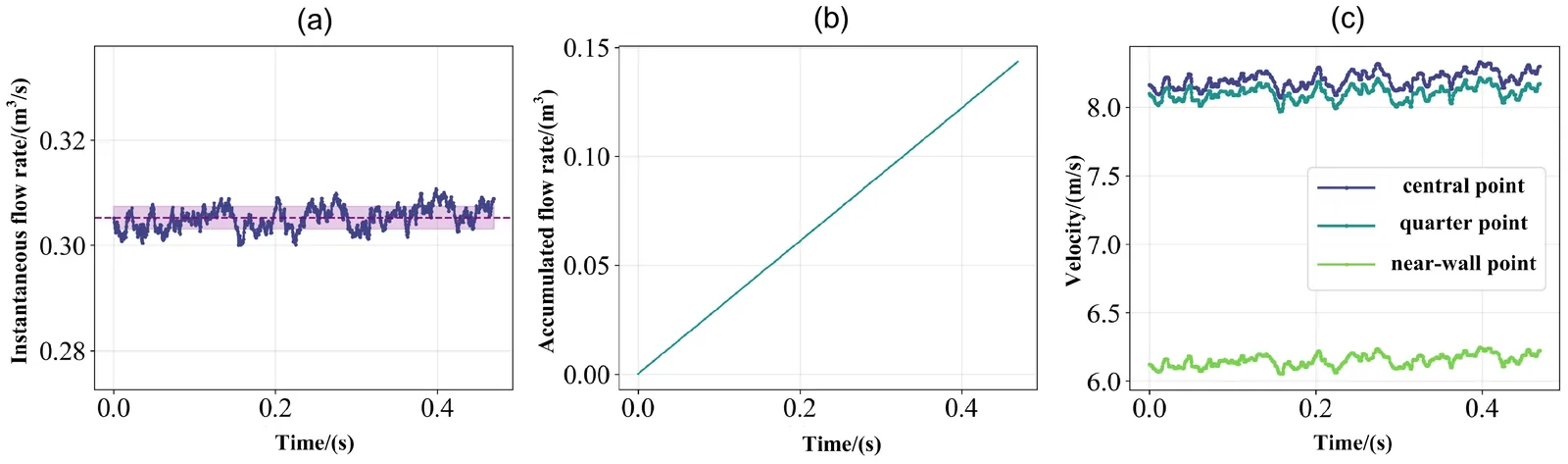
Test results of 1000 consecutive frames at 8 m/s (**a**) the instantaneous flow rate curve; (**b**) the accumulated flow rate curve; (**c**) the velocity variation curves at each measurement point.

After completing the system setup and debugging, a scanning test was first conducted over the full range of flow velocities from 1 to 10 m/s (with a step size of 1 m/s) to verify the basic functionality of the system and to observe the characteristics of its raw output data. At each stable flow velocity, the system synchronously acquired Doppler signals from the three measurement points at a sampling rate of 40 MS/s, with 1000 frames of data continuously recorded for each condition. The instantaneous velocity sequences were obtained through spectral analysis, and the velocity profile was fitted based on the three-point velocity measurements. The instantaneous volumetric flow rate was then calculated by numerical integration. Figure 10, Figure 11 and Figure 12 present the measurement results at three typical flow velocities of 2 m/s, 5 m/s, and 8 m/s.

The flow rate measurement results under the above three operating conditions preliminarily validate the stability and physical correctness of the proposed system. From Figure 10a, Figure 11a and Figure 12a, it can be observed that under all operating conditions, the calculated instantaneous flow rates fluctuate within a small range around the mean value (indicated by the purple dashed line), with the vast majority of data points falling within the range of the mean μ ± one standard deviation σ (the purple shaded area). This indicates that the single-measurement output of the system is stable under each independent operating condition, with no significant drift or abnormal jumps. From Figure 10b, Figure 11b and Figure 12b, it is evident that the accumulated flow rate curves under all operating conditions exhibit smooth, strictly linear trends with consistent slopes and no abrupt changes, which intuitively demonstrates that the numerical integration process from velocity signals to flow rate calculation is continuous, thereby confirming the reliability of the algorithmic procedure. From Figure 10c, Figure 11c and Figure 12c, under all operating conditions, the velocity at the center point is the highest, followed by that at the quarter-width point, and the velocity at the near-wall point is the lowest, with a clear hierarchical relationship maintained among the three points. Moreover, the velocity fluctuations at the three points exhibit significant temporal correlation, which is in full agreement with the theoretical velocity profile of fully developed turbulent flow, thereby proving the correctness of the measurement results from the perspective of physical principles.

**Figure 13:**
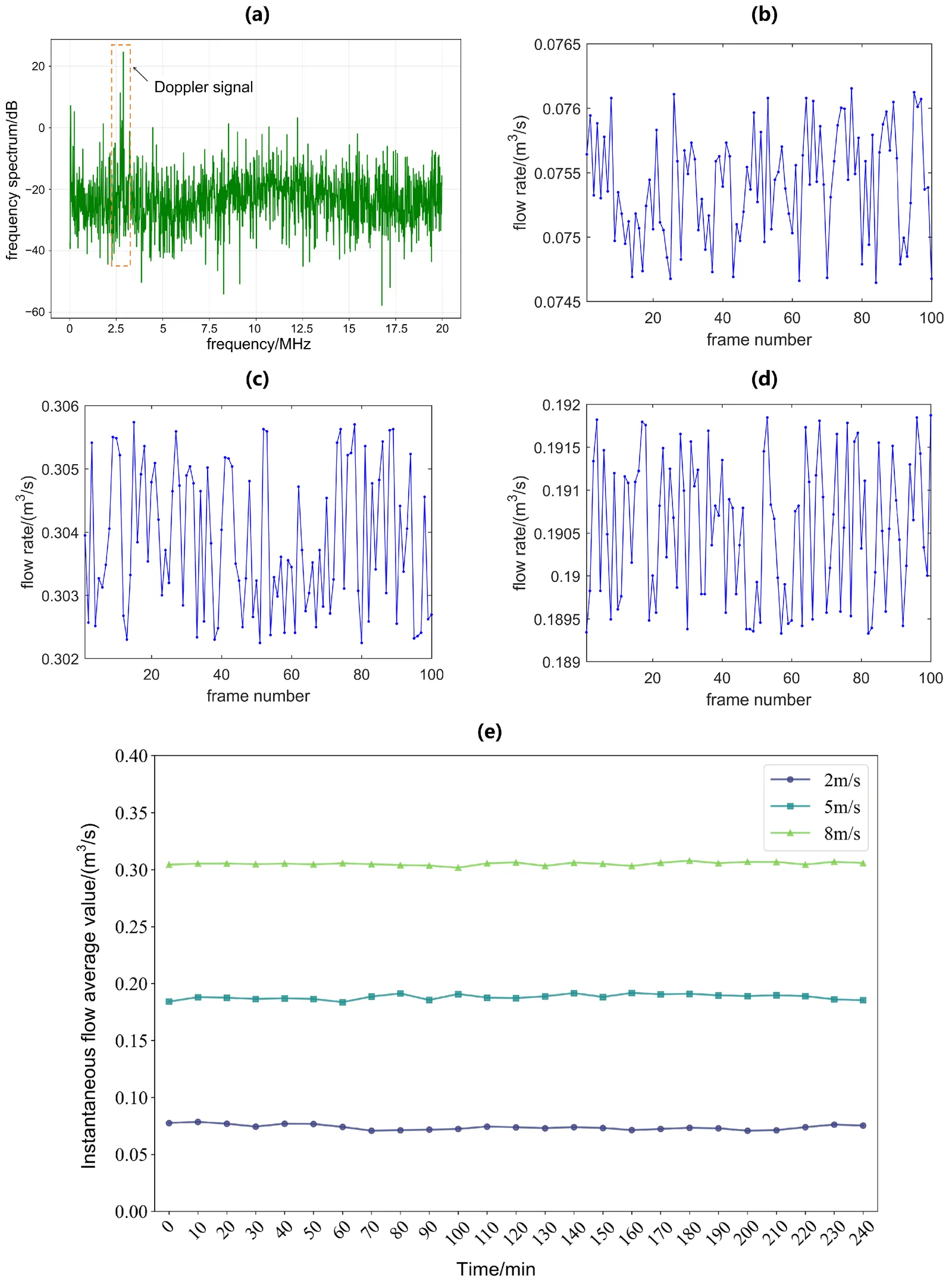
Results of system stability test (**a**) the spectrum of the Doppler signal; (**b**) results of 100 consecutive frames at 2 m/s speed; (**c**) results of 100 consecutive frames at 5 m/s speed; (**d**) results of 100 consecutive frames at 8 m/s speed; (**e**) average flow rate at different speeds.

The long-term stability of the system was assessed through experiments at three typical velocities (2 m/s, 5 m/s, and 8 m/s). Once the wind tunnel outlet velocity reached and stabilized at the setpoint, 25 data sets were recorded at 10 min intervals, with 100 frames per set, and the corresponding time-averaged instantaneous volumetric flow rates were calculated. The results are shown in Figure 13. Shown in Figure 13a is the spectrum of a Doppler signal. Figure 13b–d display the flow rates from 100 successive frames at 2, 5, and 8 m/s, respectively, while Figure 13e depicts the evolution of the averaged flow rate over 4 h at these three velocities.

As can be observed from Figure 13a, the spectral amplitude of the Doppler signal exceeds 20 dB, while that of the noise remains below 0 dB. According to the data presented in Figure 13b–d, the fluctuations in the instantaneous flow rate are 0.0015 m^3^/s, 0.0025 m^3^/s, and 0.0034 m^3^/s, respectively. From Figure 13e, it can be seen that under the three velocity settings, the time-averaged instantaneous flow rates measured at different time instants exhibit only minor fluctuations, with corresponding coefficients of variation of 2.99%, 1.19%, and 0.44% for the three test groups, respectively. Throughout the continuous 4 h operation, no significant abrupt changes or systematic drift were observed, indicating that the proposed three-point synchronous LDV measurement system possesses good stability under long-term operating conditions. The instantaneous flow rates display small random fluctuations around their respective mean values. Combined with the absence of systematic drift, this observation suggests that such fluctuations are predominantly attributable to the inherent stochastic disturbances of the measured flow field (e.g., turbulent fluctuations) and residual random noise that is difficult to eliminate entirely during measurement, rather than to accumulation of systematic errors of the system itself.

**Figure 14:**
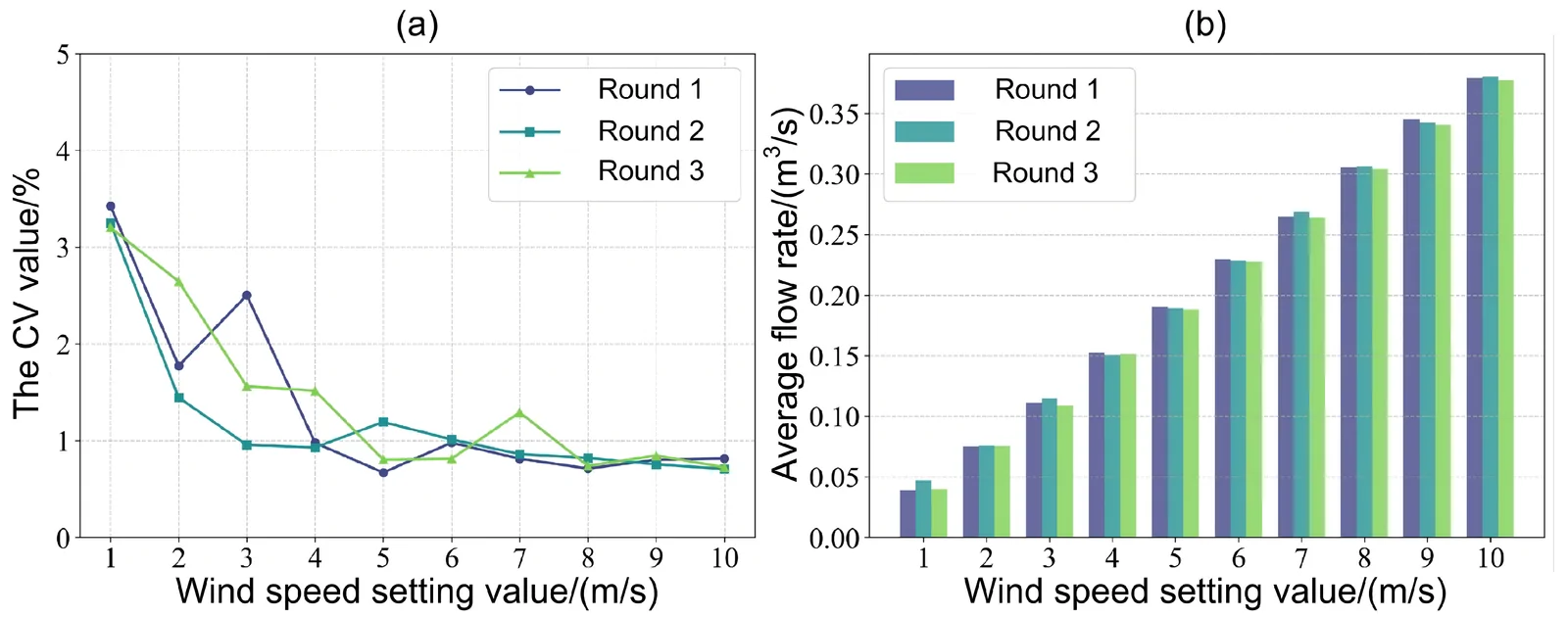
Results of system repeatability test (**a**) analysis of flow variability coefficient; (**b**) average instantaneous flow rate comparison.

Repeatability tests were conducted at ten velocities from 1 m/s to 10 m/s with 1 m/s increments, and three independent measurement runs were performed. The coefficients of variation (CV) values for each run are listed in Table 4, and the trends are plotted in Figure 14a. The results show that (i) the three CV curves closely follow the same trend with no significant drift or anomalies, confirming that the system’s single-measurement performance is consistent across runs and that no time-dependent degradation of key components occurred; (ii) the CV decreases rapidly with increasing velocity and stabilizes above 3 m/s, with most values around 1% and below 2% for 3–10 m/s, demonstrating high measurement precision in this range; and (iii) for 1–2 m/s, CV ranges from 2% to 4%, mainly because particles take longer to traverse the measurement volume at low speeds, resulting in low-frequency, low-amplitude Doppler signals and reduced SNR—a well-known limitation of LDV that nonetheless keeps CV within acceptable bounds. Run-to-run repeatability, which indicates measurement consistency over time, is a key metric for long-term stability. Figure 14b compares the mean flow rates across runs. For most velocities, the inter-run differences are small, with maximum relative deviations typically below 1.5%. The results confirm the physical correctness of the measurements, and the low inter-run variability (CV < 3.5%) across all conditions highlights the excellent long-term stability of the system.

To qualitatively verify the physical trends of the system, its measurement results were compared to those obtained by an indirect measurement method based on a single-point Pitot tube at different flow velocities. The Pitot tube obtains pipeline flow rate using a single-point measurement and fitting method. The core assumption of this method is that the ratio of the centerline velocity to the cross-sectional average velocity is a constant. The empirical formula for its flow measurement is as follows:

(7) $$Q=k\cdot A\cdot v_{\max}$$

where Q is the Pipeline flow rate; $v_{\max}$ is the center point velocity of the pipeline; k is the empirical coefficient, it is the ratio of the average flow velocity over the cross-section to the maximum flow velocity at the center, which is closely related to the Reynolds number; A is the cross-sectional area.

**Figure 15:**
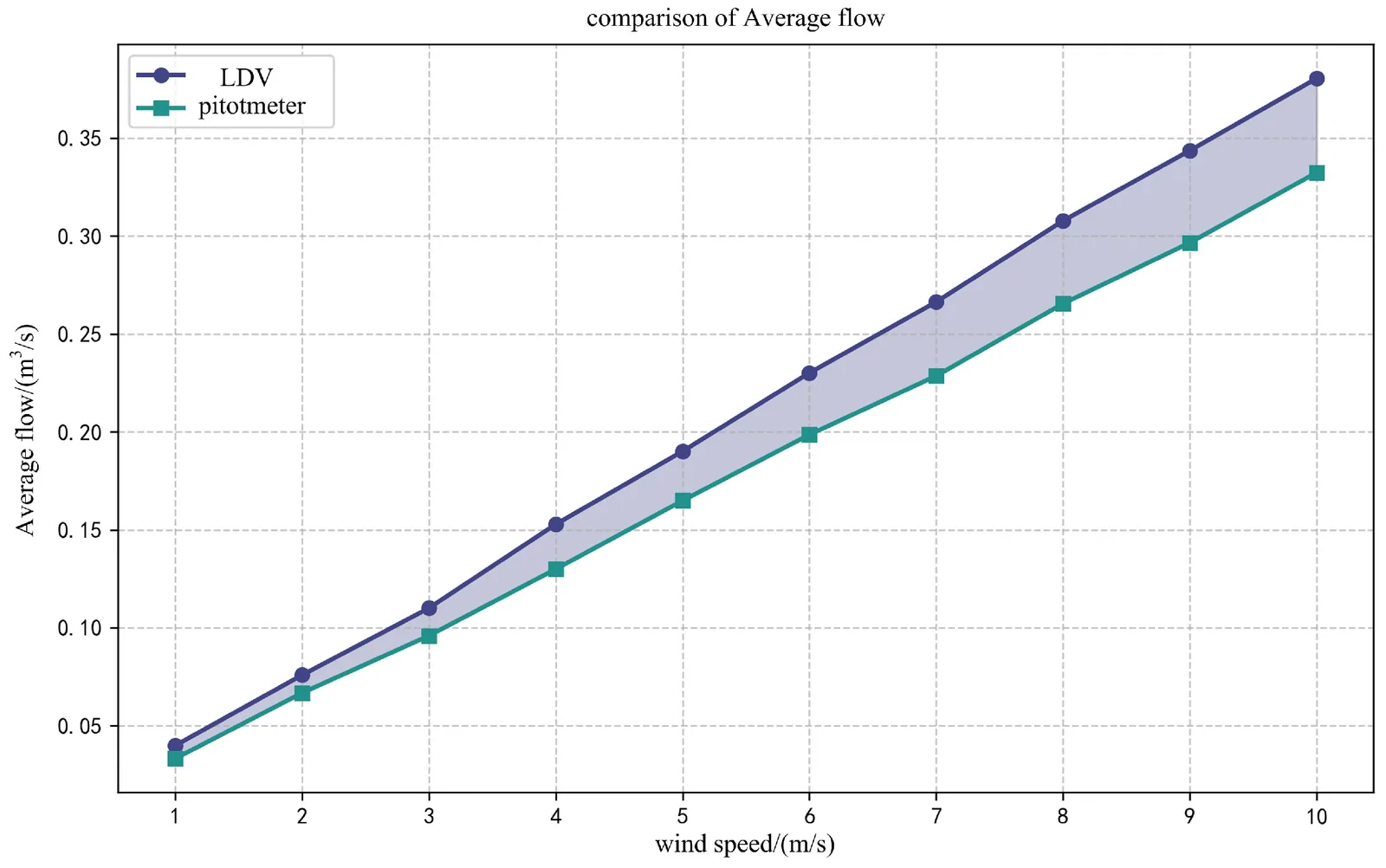
Comparison of average flow rate measurement results under different wind speed conditions.

The experimental results are presented in Figure 15. As shown in Figure 15, the flow rates measured by the proposed system and those obtained by the single-point Pitot tube exhibit essentially consistent trends with increasing flow velocity, both demonstrating a good linear relationship, which preliminarily confirms the physical reasonableness of the measurement results. In fact, the Pitot tube can only characterize the peak velocity in the central flat region, but fails to reflect the velocity gradient in the transition region or the boundary-layer attenuation pattern in the near-wall region. However, the square pipeline exhibits pronounced nonlinear characteristics with a distinct “central flat region, transitional gradient region, and steep near-wall drop region.” Consequently, the Pitot tube method, which relies on single-point measurement, cannot achieve high accuracy when applied to flow measurement in square pipelines. Therefore, as the flow velocity increases, the absolute deviation between the two methods shows a gradually expanding trend. This is primarily attributed to the fact that the Pitot tube is a single-point measurement device, which estimates the cross-sectional average velocity by measuring the velocity at the center point; the higher the flow velocity, the larger the error introduced by commonly used empirical formulas. This also indirectly demonstrates the superiority of the multi-point measurement system. Over the flow velocity range of up to 10 m/s, the average flow rate deviation in the proposed system relative to the Pitot tube measurements does not exceed 0.05 m^3^/s.

**Figure 16:**
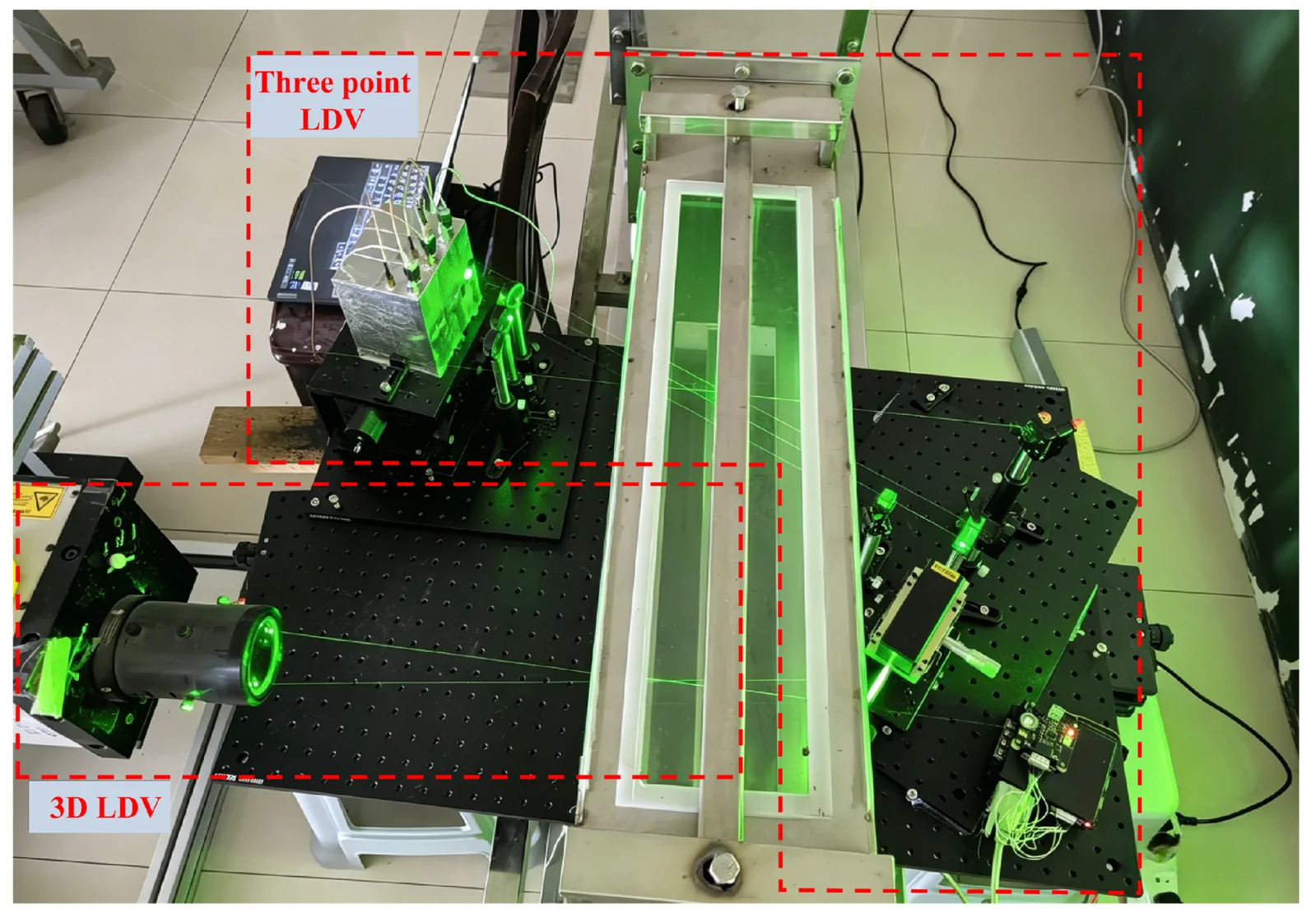
System layout of precision comparison experiment.

To quantitatively evaluate the accuracy of the proposed three-point synchronous LDV flow measurement system, a TSI three-dimensional laser Doppler velocimeter (3D LDV) was employed as the reference standard for flow measurement, with its measurement accuracy reaching as high as 0.05%. The measured values from this system were used as the flow rate benchmark for comparison. This 3D LDV system performed point-by-point scanning measurements across the entire cross-section (200 mm × 200 mm) of the wind tunnel test section through precise two-dimensional scanning, with a point spacing of 2 mm, thereby obtaining time-averaged velocities at tens of thousands of spatial points. By integrating the resulting dense velocity field over the area, the volumetric flow rate through the cross-section could be calculated. The two devices were installed at different positions along the wind tunnel test section, allowing both systems to measure the same steady flow field without mutual interference. The layout of the comparative experiment is illustrated in Figure 16.

**Figure 17:**
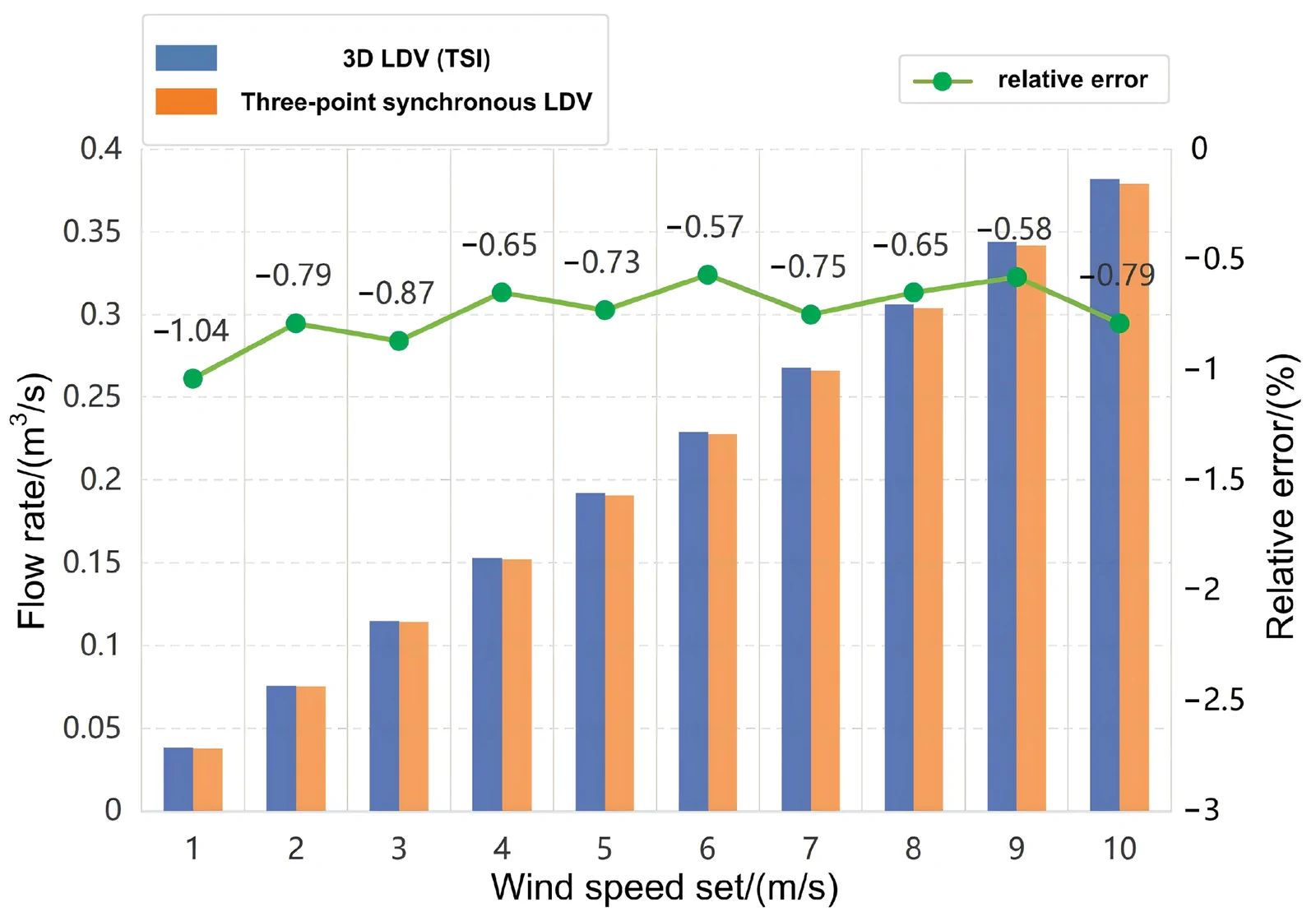
Flow rate measurement results and relative errors.

In the experiments, the wind tunnel was operated at ten setpoints across a velocity range of 1 m/s to 10 m/s, with an interval of 1 m/s, for comparative testing. At each steady-state operating condition, the instantaneous average flow rate $Q_{3\text{-}point}$ measured by the three-point synchronous LDV system and the reference flow rate $Q_{3D\_LDV}$ obtained by the 3D LDV scanning system (TSI Inc., Shoreview, MN, USA) were recorded simultaneously. Using $Q_{3D\_LDV}$ as the reference value, the relative measurement error of the proposed system was calculated by $\delta =\left(Q_{3\text{-}point}-Q_{3D\_\mathrm{LDV}}\right)/Q_{3D\_\mathrm{LDV}}\times 100\%$. The results are presented in Figure 17, which demonstrate that the measurements obtained by the proposed system are in excellent agreement with the reference values at all ten characteristic velocities, with relative errors ranging from −1.04% to 0 and a mean relative error of −0.74%. The measurement errors of the system can be attributed to six main factors: (1) optical alignment errors—minor deviations in the angle and position of optical components are unavoidable during installation; (2) spatial positioning errors of the measurement points—influenced by lens mounting errors, focal length inaccuracies, and the thickness and refractive index of the wall window, which propagate into radial coordinate errors in the reconstructed profile; (3) flow field errors from the wind tunnel—arising from non-uniformities and instabilities in the test section; (4) electronic noise in the data acquisition chain, including APD dark current noise, amplifier noise, and quantization noise, which adds to the Doppler signal and increases the uncertainty of velocity extraction; (5) signal processing errors—limited by the inherent resolution and spectral estimation accuracy of the algorithm, especially under low SNR, where frequency estimates may fluctuate about the true value, resulting in greater data scatter; and (6) fitting errors—due to the discrepancy between the fitted velocity profile model and the true flow field. The proportions of each error factor are shown in Table 5.

The experimental results confirm that the proposed three-point synchronous LDV system is not only feasible in principle but also possesses the measurement accuracy required for practical engineering applications. Moreover, in contrast to the 3D LDV system, which requires several hours of full cross-section scanning for a single operating condition, the proposed system achieves instantaneous capture of the flow field velocity through synchronous three-point detection, thereby improving the measurement efficiency to the second level for a single operating condition. This effectively addresses the poor temporal efficiency inherent in conventional point-by-point scanning methods and demonstrates significant engineering application value. A comparison of the proposed method with conventional approaches (Pitot tubes, single-point LDV, and scanning LDV) is summarized in Table 6, covering accuracy, cost, system complexity, and measurement time. It can be seen that Pitot tubes and single-point LDV are primarily limited by their insufficient accuracy, while scanning LDV is constrained by its complex structure, high cost, low efficiency, and unsuitability for time-varying flows. By contrast, the multipoint synchronous LDV method proposed in this work features high accuracy, a simple system architecture, and rapid flow-field reconstruction.

## 5. Conclusions

In this paper, based on the dual-beam differential laser Doppler velocimetry technique, a gas flow rate measurement method for square pipelines is proposed, which integrates “three-point synchronous measurement, profile fitting, and symmetric integration.” A three-point synchronous LDV measurement system is designed and constructed, featuring a transmitting optical path based on the “three beam splitters and two mirrors” scheme and a receiving optical path based on the “multi-lens independent reception” scheme. Systematic tests and validations are conducted for square pipeline applications. The system operates stably with good measurement repeatability. In comparison with the flow calculation method using a single-point Pitot tube combined with an empirical formula, it is revealed that the single-point method relying on a fixed empirical coefficient introduces significant model errors in square pipelines. In contrast, the proposed method, which directly fits the velocity profile and integrates it for flow calculation, effectively circumvents such errors and is inherently more accurate and universally applicable in principle. In practice, the velocity distribution in a square duct varies with position and exhibits distinct regional differences. The measurement points selected by the three-point synchronous measurement method are highly representative, covering different characteristic regions and capturing the variation pattern of the velocity across the entire cross-section. This approach therefore significantly reduces the error in flow field reconstruction. Moreover, simulation results of the flow field distribution reveal that the velocity profiles differ with varying flow velocities, particularly in the near-wall region. Consequently, the conventional empirical correction factor method, which applies a fixed correction factor, inevitably leads to increased errors under different velocity conditions. Through comparison with the TSI reference standard (3D LDV), the measurement results of the proposed system are in close agreement with the reference values, with relative errors all below −0.8%. These results demonstrate that the proposed system is practically feasible for pipeline gas flow rate measurement. Compared to conventional flow measurement devices such as Pitot tubes and electromagnetic flowmeters, the system developed in this work offers higher measurement accuracy. Nevertheless, it suffers from several limitations: (a) both ends of the pipe must be optically transparent; (b) seeding particles must be introduced into the fluid; and (c) the overall system configuration is relatively complex. In future work, a two-dimensional flow field reconstruction technique can be developed based on sparse measurement point data combined with intelligent algorithms such as deep learning. By training neural network models to predict the velocity distribution over the entire cross-section with high accuracy from limited-point velocity data, the flow measurement accuracy and system adaptability under complex flow conditions can be further enhanced.

## Figures and Tables

**Table 1:** The main features of several common flow measurement devices.

Measuring Equipment	Operating Principle	Typical Disadvantages	Typical Disadvantages
Pitot tube	Bernoulli’s principle	The structure is extremely simple and the cost is low; it can withstand high temperatures and pressures and is suitable for use with steam and air flow.	It measures the velocity of individual points, indicating a large error for the entire pipe (with an accuracy of approximately 3%); it is prone to clogging, and the small differential pressure at low flow rates leads to poor accuracy.
Electromagnetic flowmeter	Faraday’s law of electromagnetic induction	No flow obstruction, low pressure loss; high precision (±0.5%); unaffected by temperature/density/viscosity; especially suitable for dirty or corrosive liquids containing particles.	It can only measure conductive liquids (with conductivity > 5 μS/cm), and is unable to measure oil, pure water or gases.
Vortex street flowmeter	The principle of the vortex street: the frequency is directly proportional to the flow velocity.	The applicable medium is wide (including liquids, gases and vapors); there is no zero-point drift; the structure is simple and the range ratio is wide.	Poor vibration resistance, sensitive to pipeline vibrations; not suitable for measuring high-viscosity, low-flow-rate, small-diameter pipes (below DN300).
Turbine flowmeter	Fluid drives the impeller to rotate: the rotational speed is directly proportional to the flow velocity.	One of the highest levels of precision (±0.2% to ±0.5%); good repeatability and fast response.	It has mechanical moving parts, which are prone to wear; it has poor anti-pollution ability and requires the medium to be clean.
Ultrasonic flowmeter	Ultrasonic time difference method/Doppler effect	Non-contact measurement (external clamp type, can be installed without production halt); no pressure loss; capable of measuring strong corrosive and non-conductive media; particularly suitable for large pipe diameters.	High requirements for the purity of the medium (turbidity > 10 kg/m^3^, with large error); affected by the material of the pipe wall and scaling, the accuracy is reduced; not suitable for non-metallic pipes.

**Table 2:** Comparison of fitting performance of different functions.

Type of Fitting Function	Fit Goodness R^2^	Average Relative Error (%)
power-law function	0.949	9.39
logarithmic function	0.976	7.74
polynomial function	0.863	17.10
arctangent function	0.986	4.69

**Table 3:** Main technical parameters of BLWT-200/200 DC.

Parameter	Values	Unit
Type of construction	Single working section direct current type	\
Work section size	200 × 200	mm
Flow rate range	0.2~40	m/s
Airflow uniformity	≤1	%
Airflow stability	≤0.5	%
Turbulivity	≤0.5	%
Airflow deflection angle	≤0.5	°
Motor power	380	kW
Wind tunnel structure dimensions	4.35 × 1.11 × 1.8	m

**Table 4:** The CV values under different wind speed settings in the three-round experiment.

Setting Speed (m/s)	CV of Round 1 (%)	CV of Round 2 (%)	CV of Round 3 (%)
1	3.43	3.25	3.20
2	2.77	2.39	3.06
3	2.64	1.34	2.14
4	1.35	1.23	1.68
5	0.86	1.25	0.89
6	1.25	1.09	0.91
7	1.01	0.94	1.33
8	0.86	0.91	0.93
9	1.26	1.74	1.00
10	0.90	1.11	0.80

**Table 5:** The proportion of each error.

Factor	Proportion (%)
optical alignment	<10
spatial positioning	≈15
flow field non-uniformities	≈30
electronic noise	≈25
signal processing	<15
curve fitting	≈5

**Table 6:** A comparative analysis of the main parameters of this method and those of the existing methods.

Measuring Method	Precision	System Complexity	Cost	Measurement Efficiency
Pitot tube	low accuracy	simple	low	fast
Single-point LDV	medium accuracy	simple	moderate	fast
Scanning LDV	high accuracy for stable flow field low accuracy for varying flow field	complicated	high	slow
Multiple-point synchronous LDV	high accuracy	simple	moderate	fast

## Data Availability

Data is contained within the article.
